# Optical microangiography reveals temporal and depth-resolved hemodynamic change in mouse barrel cortex during whisker stimulation

**DOI:** 10.1117/1.JBO.25.9.096005

**Published:** 2020-09-17

**Authors:** Adiya Rakymzhan, Yuandong Li, Peijun Tang, Ruikang K. Wang

**Affiliations:** University of Washington, Department of Bioengineering, Seattle, Washington, United States

**Keywords:** optical coherence tomography, optical microangiography, intrinsic optical signal imaging, cerebral blood flow, hemodynamic response, neurovascular coupling

## Abstract

**Significance:** Cerebral blood flow (CBF) regulation at neurovascular coupling (NVC) plays an important role in normal brain functioning to support oxygen delivery to activating neurons. Therefore, studying the mechanisms of CBF adjustment is crucial for the improved understanding of brain activity.

**Aim:** We investigated the temporal profile of hemodynamic signal change in mouse cortex caused by neural activation and its variation over cortical depth.

**Approach:** Following the cranial window surgery, intrinsic optical signal imaging (IOSI) was used to spatially locate the activated region in mouse cortex during whisker stimulation. Optical microangiography (OMAG), the functional extension of optical coherence tomography, was applied to image the activated and control regions identified by IOSI. Temporal profiles of hemodynamic response signals obtained by IOSI and OMAG were compared, and OMAG signal was analyzed over cortical layers.

**Results:** Our results showed that the hemodynamic response to neural activity revealed by blood flow change signal signal through IOSI is slower than that observed by OMAG signal. OMAG also indicated the laminar variation of the response over cortical depth, showing the largest response in cortical layer IV.

**Conclusions:** Overall, we demonstrated the development and application of dual-modality imaging system composed of IOSI and OMAG, which may have potential to enable the future investigations of depth-resolved CBF and to provide the insights of hemodynamic events associated with the NVC.

## 1 Introduction

Neurovascular coupling (NVC) is associated with a regional increase in the cerebral blood flow (CBF) during local neural activation, also referred to as functional hyperemia.^1^ NVC is a vital process, which regulates oxygen delivery through blood stream to supply the activated neurons for normal brain functioning.^1^ Therefore, studying the mechanisms of CBF regulation at NVC is crucial for the improved understanding of brain activity under normal and neurodegenerative conditions.

Intrinsic optical signal imaging (IOSI) serves as a well-established method in evaluating hemodynamics and oxygenation during neural activity.^2^^–^^4^ IOSI allows for the extraction of reflectance signal changes caused by tissue scattering and absorption alterations of intrinsic chromophores such as hemoglobin, or the changes in cerebral blood volume.^5^ The given reflectance method demonstrated excellent spatial and temporal correspondence with calcium-sensitive fluorescence dye imaging.^6^ Although the technology is widely used, the detection with IOSI is two-dimensional and limited in its resolution, hence has difficulty in providing layer-specific cortical information.

The variation of neuronal density among six cortical layers results in different metabolic demands, and consequently, different blood flow adjustments.^7^ Accordingly, several studies have demonstrated the variation of hemodynamic response to neural activation over cortical depth. Particularly, the studies with functional magnetic resonance imaging showed the change of blood oxygenation level-dependent signal amplitude over cortical layers.^8^^,^^9^ The fastest vessel dilation in the deepest measured arterioles and capillaries (∼550  μm) was revealed with two-photon microscopy.^9^ Another analysis on capillaries showed the most pronounced changes in red blood cells (RBCs) velocity and flux beyond the depths of 200  μm.^10^ In the research study with dynamic contrast optical coherence tomography (OCT), the shortest mean transit time and capillary transit time heterogeneity were observed in the layers 4 and 5.^11^ Despite all the experimental findings and explorations, there is still a lack of depth and temporal information on hemodynamic variation across the cortex. This challenge calls for *in-vivo* depth-resolved, three-dimensional (3-D) functional imaging of CBF with a high spatiotemporal resolution.

OCT is a noninvasive optical technology, which in contrast to other microscale-resolution imaging modalities, enables high-resolution 3-D visualization and quantification of internal structures in biological tissues up to 2 mm beneath the tissue surface.^12^ In the recent advancement of OCT–optical microangiography (OMAG), the dynamic complex OCT signals from moving RBCs are separated from the static tissue signals to enable the visualization of functional microvascular networks permeating the tissue beds.^13^ In previous studies, OMAG was applied to measuring the capillary blood flow in human skin,^14^ gingiva,^15^ retina and choroid,^16^ and mouse cortex.^17^ OMAG also allowed for measuring the velocity and transit time of capillary RBCs in mouse somatosensory cortex.^18^ However, these studies have examined neither the temporal profile of hemodynamic change in response to neural activation nor the variation of depth-resolved blood flow changes.

In this study, we employ OMAG and IOSI to image the activated somatosensory cortex of mouse brain. Temporal profiles of both signals are compared, and depth differentiated OMAG blood flow changes during functional activation are observed and discussed to provide additional insight of hemodynamic regulation at the NVC.

## 2 Materials and Methods

### 2.1 Animal Preparation

Animal surgeries and experimental procedures in this study were approved by the Institutional Animal Care and Use Committee (IACUC) of the University of Washington and conducted in accordance with University of Washington guidelines and ARRIVE guidelines. C57BL/6 mice (Charles River Laboratories, n=6, 3-month-old, 23 to 25 g) were anesthetized with a ketamine–xylazine cocktail (0.1  ml/25  g mouse, IP). Physiological parameters such as adequate anesthesia depth (no hindpaw reflexes) and body temperature (36.8±0.2°C) were monitored throughout all experimental procedures. Optical access to the somatosensory cortex was obtained through a cranial window surgery in the left cerebral hemisphere according to protocol described in Ref. 19. In brief, after the skin and meninges tissues were removed from the skull surface, a window opening was drilled in the skull and the exposed brain was sealed with a 5-mm circular glass coverslip using glue.

### 2.2 Intrinsic Optical Signal Imaging

**Fig. 1:**
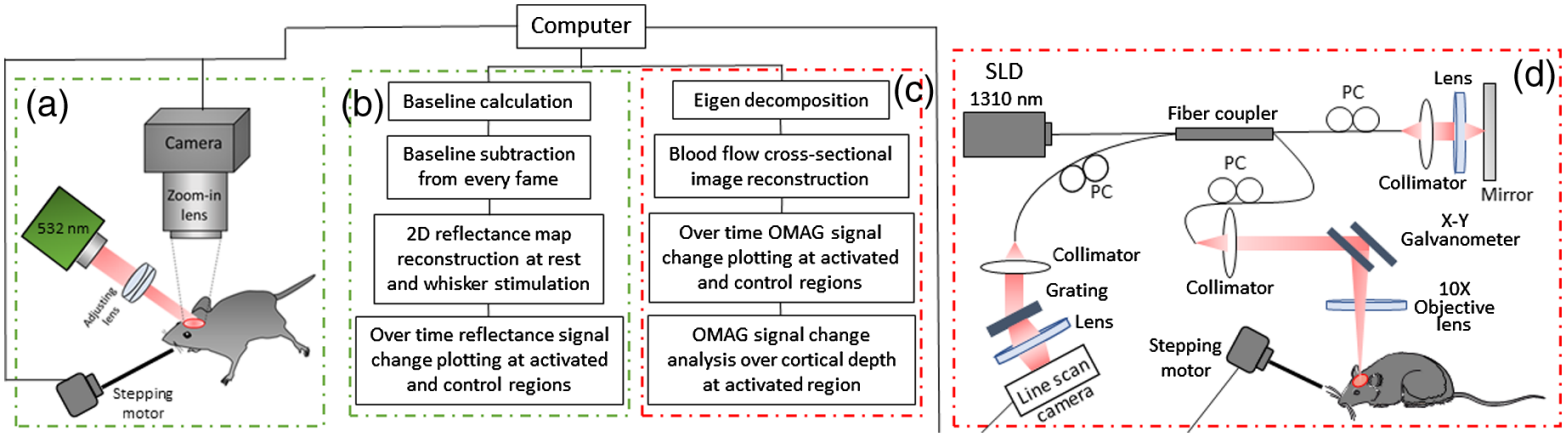
Schematic diagram of IOSI and OCT systems and data processing. (a) IOSI system setup, (b) IOSI data processing steps, (c) OMAG data processing steps, and (d) OCT system setup. SLD, superluminescent diode; PC, polarization controller.

IOSI was performed to spatially locate the hemodynamic response in mouse cortex. In IOSI setup [Fig. 1(a)], a 532-nm (50 mW) laser source (MGL-III-532, CNI) illuminated the cranial window at an approximate angle of 30 deg. The choice of wavelength was based on previous experimental studies where the isosbestic points of deoxygenated (Hb) and oxygenated hemoglobin (HbO) of 530 to 565 nm were used to provide a high-contrast reflectance signal of blood volume changes in the activated cortex area. The given spectral range allows an equal absorption of Hb and HbO and thus makes the measurement independent of oxygen variations. The 532-nm wavelength was so chosen for the sensitive measurement of hemoglobin (HbT) content in the total blood volume.^5^ A research-grade area-scan camera (A404km-RT, BaslerAG) was used to detect the reflectance light from the brain with the acquisition rate of 15 Hz. The exposure time of the camera was set to 40 ms and the frame size was 1280×1024  pixels representing 5.5×5.5  mm of cortical area. In total, 450 frames were acquired within 30 s.

Although the imaging system setup is similar to a laser speckle contrast imaging (LSCI) system setup, as it uses a coherent light source (laser diode), the processing pipeline used in our study fits rather IOSI than LSCI. A baseline was obtained by temporal averaging of the first 10 frames. The baseline was then subtracted from every frame, and the resulting frames were normalized to the baseline. Two-dimensional reflectance images and time-lapsed reflectance signal profiles at both activated region (R1) and the nonactivated regions (R2) were presented. At each region, the change in reflectance signal (−ΔR/R) was averaged across six animals. Hence, we measured the changes in the reflectance of tissue, while in LSCI, the changes in the speckle pattern of light impinging on tissue are tracked.

### 2.3 Optical Microangiography

OMAG scans were obtained using spectral-domain OCT (SD-OCT) system [Fig. 1(d)]. In this system, a superluminescent diode (SLD) (LS2000B, Thorlabs Inc.) with a center wavelength of 1310 nm and a spectral bandwidth of 110 nm was used as a light source and provided ∼7.5-μm axial resolution in air (∼5.1  μm in mouse brain tissue). The system consisted of sample and reference arms. Divided by a 2×2 optical coupler, one part of SLD light passed through the optical system composed of a collimator, X-Y galvanometer, and a 10× objective lens with 10-μm lateral resolution, forming the sample arm. The rest of the light was directed into another optical system with a collimator and a lens to form the reference arm. An optical coupler integrated the first part of the light backscattered from the sample and the second part backscattered from the mirror. Next, to detect the interference signal, the output light was transmitted to a spectrometer with a spectral resolution of 0.141 nm, which provided 2.22 mm of imaging depth into the sample. The spectrometer was equipped with an InGaAs line scan camera (SUI, Goodrich Corp.) capable of 92-kHz A-line rate to capture the interference signal.

The scanning protocol was the repeated B-scans with a scan rate of 300 fps. Every B-scan was formed by 200 A-lines at a rate of 92 kHz. In total, 9000 frames were acquired within 30 s, covering an approximate cross-sectional region of 1.4  mm (lateral)×2.22  mm (depth) with a uniform transverse sampling of 7.5  μm/pixel. Imaging was performed at activated and control regions identified by IOSI during rest and stimulation periods.

The detailed algorithm description of OMAG analysis [Fig. 1(c)] based on eigen-value decomposition can be found in Ref. 20. In short, the signals from moving particles were separated from the static tissue signals using eigen-regression filter based on the energy of each eigenvalue. The OCT signal at each imaging voxel was considered as a superposition of three independent components: clutter (static and slowly moving tissue structures), blood flow signal (moving RBCs), and noise (system and shot noise). The given signal was presented in the form of correlation matrix, where the clutter components were decomposed into eigenvectors and eigenvalues. The first two highest power eigenvalues corresponding to tissue bulk motion were excluded from the covariance matrix among the repeated B-scans to produce blood flow OMAG images. Cross-sectional OMAG images and signal change time profiles at both activated region (R1) and the nonactivated regions (R2) were then demonstrated. For the time profiles, the same strategy as for IOSI was applied: a temporally averaged baseline over first 10 frames was subtracted from every frame, and the resulting frames were normalized to the baseline. The analysis of OMAG signal changes over cortical depth was also presented. The OMAG intensity signal was averaged across six animals.

### 2.4 Stimulation

Mouse whiskers were mechanically stimulated with a wood stick attached to a stepper motor (Zyltech 17HD48002H-22B). A wood stick was positioned 5 to 7 mm from the mouse face. The motor operating at a rate of 3 Hz was synchronized with both imaging systems. The motor was triggered at the 5th s of data acquisition and continued for 10 s. Each trial was 30 s in total, including 5 s of rest, 10 s of stimulation, and 15 s of poststimulation rest. There was one trial per each animal.

## 3 Results

### 3.1 Reflectance Signal Change upon Functional Activation

**Fig. 2:**
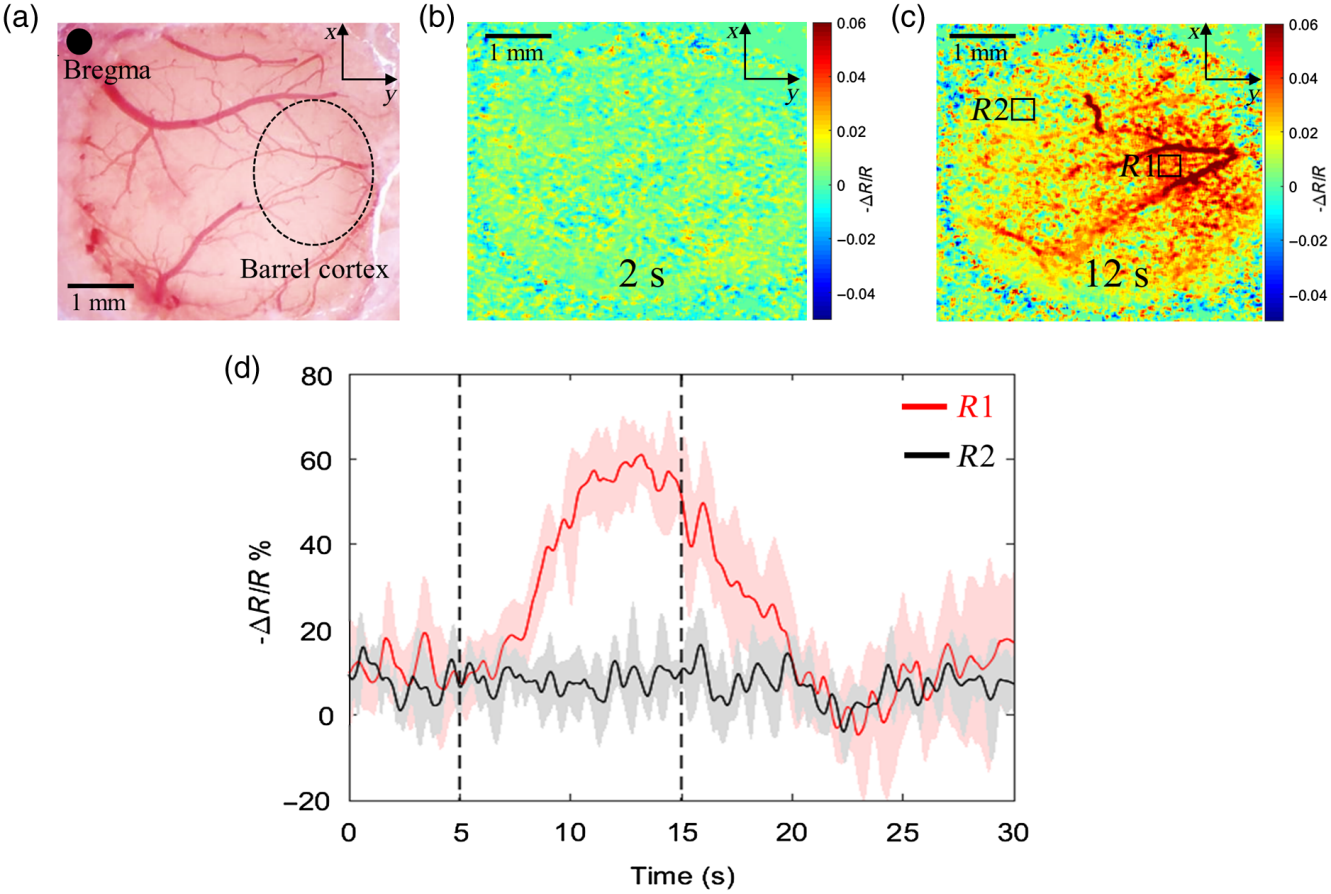
IOSI identifies the activation region in the brain cortex upon whisker stimulation. (a) Microscope image of cranial window at somatosensory cortex. (b) Reflectance map of cranial window area at resting (2nd s). (c) Reflectance map of cranial window area at stimulation (12th s). R1 indicates the activated region and R2 indicates the control region. (d) Temporal profile of reflectance signal change in response to whisker stimulation at activated (red curve) and control (black curve) regions. ΔR is a change in reflectance signal, R is a baseline reflectance signal. Dashed lines indicate the start and the end of stimulation time.

To locate the barrel cortex area, first, we imaged the cranial window [Fig. 2(a)] with IOSI before (0 to 5 s), during (5 to 15 s), and after (15 to 30 s) whisker stimulation. Figure 2(b) shows the reflectance image at the resting state (2nd s from the onset of imaging) and Fig. 2(c) shows the reflectance image at the functional activation (12th s). The latter demonstrates the localized increase of signal reflectance during whisker stimulation at the region R1, which corresponds to barrel cortex,^21^ and no pronounced change of reflectance at the region R2 (which was thus considered as the control in this study). The variation of the reflectance signal over time at the activated (R1) and nonactivated (R2) regions averaged over six animals is presented in Fig. 2(d). The dashed line indicates the start (5th s) and the end (15th s) of the stimulation period. The reflectance signal curve at the region R1 shows an obvious response peak, starting to rise in about 1 s since the onset of stimulation. It peaks at ∼12th s (about 60% increase from the baseline) then gradually decreases. There is a very slight response at nonactivated region R2 following the same trend. Here, we demonstrated the ability of our IOSI system to accurately detect the spatial and temporal reflectance response to the functional activation. The given method confirmed the stimulus-evoked activation at barrel cortex and provided further guidance for studying hemodynamic response in mouse cortex using OMAG.

### 3.2 OMAG Signal Change upon Functional Activation

**Fig. 3:**
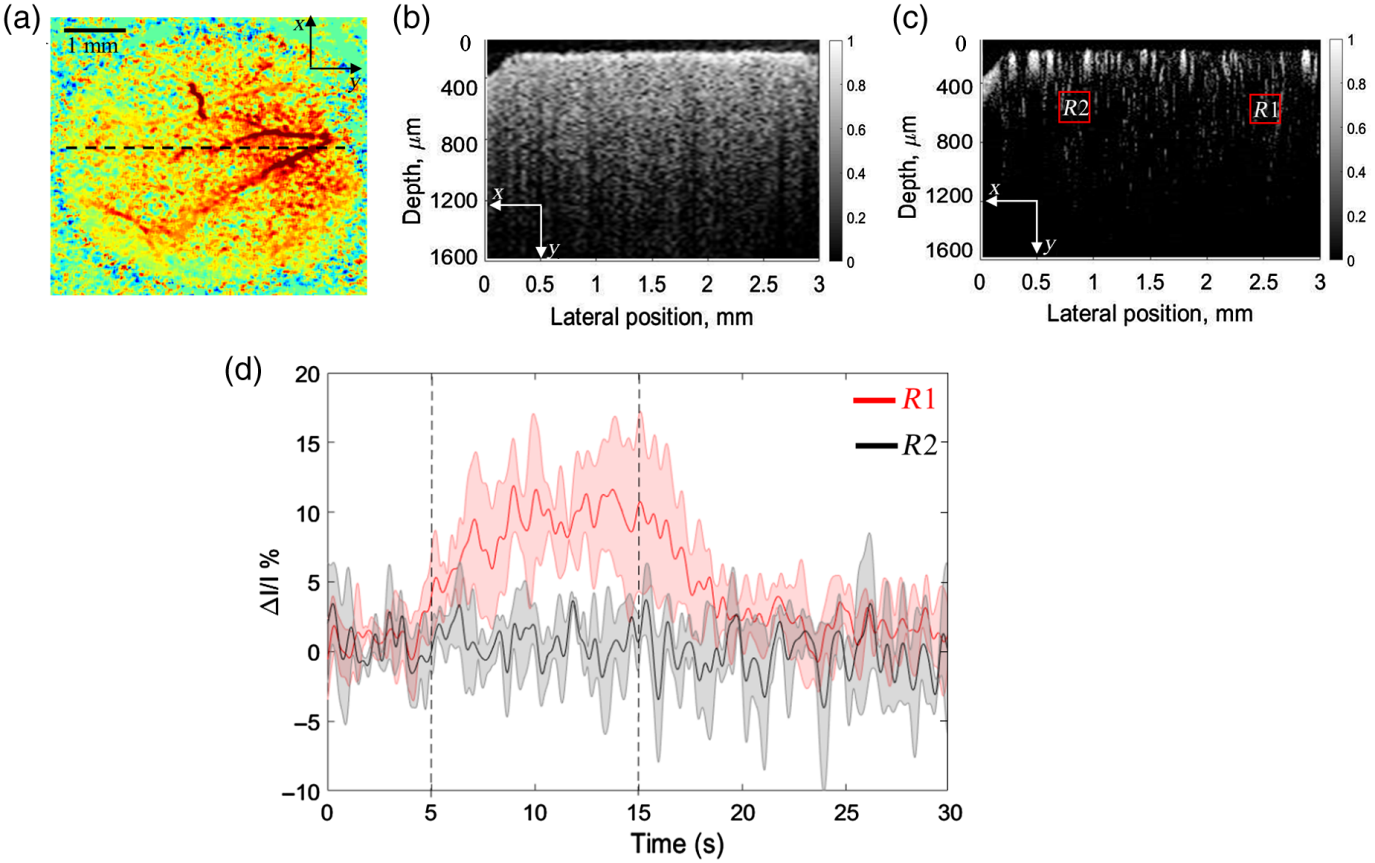
OMAG provides an ability to detect the localized hemodynamic response within the brain cortex upon whisker stimulation. (a) Reflectance IOSI map of the cortical tissue at cranial window, where the dashed line indicates the cross-section position for OCT imaging. (b) Cross-section of OCT structural image. (c) Cross-section of OMAG blood flow image. R1 indicates the activated region and R2 indicates the control region. (d) Temporal profile of OMAG signal change in response to whisker stimulation at activated (red curve) and control (black curve) regions. ΔI is the change in OMAG signal, and I is the baseline OMAG signal. Dashed lines indicate the start and the end of stimulation time.

Guided by reflectance maps obtained by IOSI above, we imaged mouse cortex with OCT at the location indicated by the dashed line in Fig. 3(a). As a result, cross-sectional OCT image of cortex structure is shown in Fig. 3(b) and cross-sectional OMAG image of cortex blood flow is shown in Fig. 3(c). Temporal profiles of OMAG signals averaged over six animals were obtained from activated (R1) and control (R2) regions [Fig. 3(d)]. There is a clear reaction of OMAG signal intensity due to functional activation, which lasted from 5th to 15th s. The response started within 1 s once the whisker stimulation began and reached its maximum (increase of ∼12%) in about 5 s. The intensity dropped once stimulation ended and eventually ran into plateau at about 20th s. These results showed that OMAG is a useful tool for time course investigation of hemodynamic change in response to neural activation.

### 3.3 OMAG Signal Variation over Depth

**Fig. 4:**
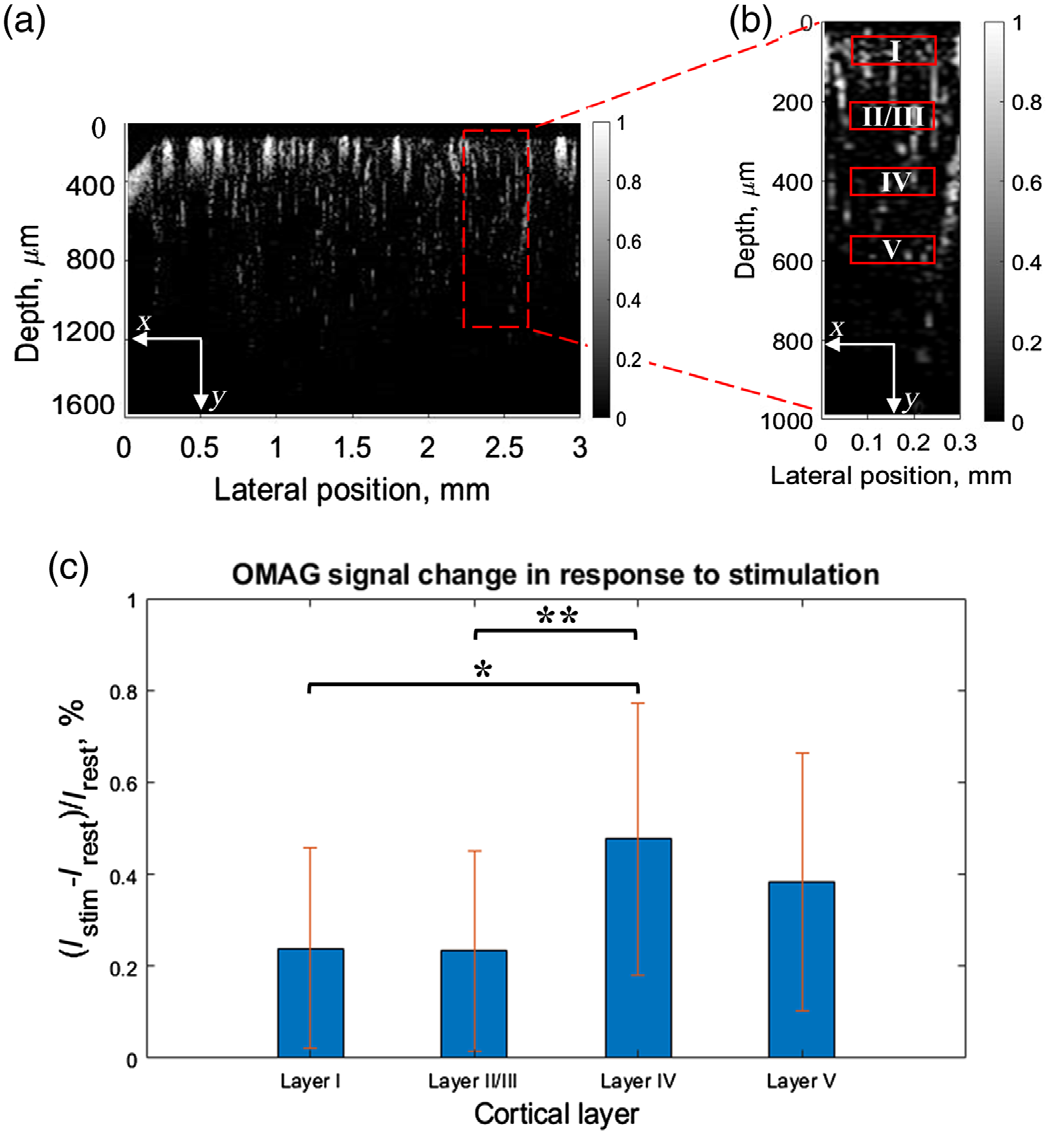
OMAG provides an ability to detect the variation of hemodynamic response over cortical depth upon whisker stimulation. (a) OMAG cross-sectional blood flow image with activated region indicated (red rectangle). (b) OMAG cross-sectional blood flow image of activated region indicated in A with layers’ location indicated. (c) Difference in OMAG signal intensity between rest and functional activation states over cortical depth averaged over six animals. Error bars represent the standard deviation. *p-value<0.02, **p-value<0.03.

Next, we examined the change of the OMAG signal over cortical depth at activated region. Figure 4(a) shows the selected cross-sectional region and Fig. 4(b) shows the approximate location of selected layers I, II/III, IV, and V, corresponding to 50 to 100  μm, 185 to 235  μm, 360 to 410  μm, and 500 to 550  μm, respectively.^22^ First, OMAG signal was spatially averaged over every layer, and temporally averaged over the time periods before and during stimulation. The average of the signal intensity before stimulation ($I_{\text{rest}}$) was subtracted from the average of the signal during stimulation ($I_{\text{stim}}$) and normalized by the average of signal intensity at rest ($I_{\text{rest}}$). This gave the percent value of OMAG signal change in response to neural stimulation. The obtained value was averaged over six mice and plotted in bar graph in Fig. 4(c). Interestingly, the largest difference in intensity between resting and stimulation states was revealed in layer IV. One-way ANOVA followed by Tukey’s honestly significant difference test for multiple comparisons was performed to assess how significantly the OMAG signal difference varied across the layers. The statistical results showed a significant difference between layers I and IV (p-value=0.02), layers II/III and IV (p-value=0.03). This experimental finding demonstrates that OMAG could be a useful tool to detect the layer-specific hemodynamic changes.

## 4 Discussion

CBF adjustment at the NVC is crucial for normal brain functioning since it provides sufficient oxygen delivery to activating neurons. Hemodynamics-based imaging techniques made a significant contribution to the understanding of the CBF adjustment. In previous studies, both IOSI and OMAG demonstrated their feasibility to visualize the CBF and quantitatively assess its dynamics.^4^^–^^6^^,^^17^^,^^18^ However, the dual application of these two techniques in studying brain functional activation has not been investigated yet. IOSI offers an excellent detection of reflectance signal from CBF alterations during neuroactivity and provides a rapid mapping over a wide field of view. Despite the given advantages, IOSI is limited to the surface layer assessment excluding the contribution of capillaries and arteries at deeper layers. In contrast, OMAG provides fast microscale visualization and quantification of CBF up to 2 mm beneath the cortex surface. However, the OMAG field of view is limited partially because the probe beam is raster-scanned to form the 3-D image of the scanned tissue volume. Used together, IOSI and OMAG can provide complementing spatial and temporal hemodynamic information during neural activity both at the surface and the depth of cortex.

In this study, we used IOSI and OMAG to image the barrel cortex of mouse brain. Our IOSI system accurately detected the spatial and temporal reflectance response to whisker stimulation, confirming the stimulus-evoked activation at barrel cortex. Guided by IOSI reflectance maps, OMAG was able to provide temporal profile of intensity signal change caused by functional activation at activated and nonactivated regions of mouse cortex. Our results demonstrated ∼60% increase of IOSI signal in response to neural stimulation, while the OMAG signal rose by only ∼12% [Figs. 2(d) and 3(d)]. Such a difference in response signal magnitude may be explained by the difference in the signal sources. IOSI detects the reflectance signal from blood flow changes, which could include the absorption by HbT, scattering inside the tissue, and residual blood flow effect. Although OMAG is sensitive to the movement of all tissue components, the cellular movement is of different order and strength compared to the blood cell movement. The imaging protocol reported in this study was optimally designed for imaging blood flow rather than cellular movements. Therefore, we believe that the measured OMAG signal changes are dominated by the vascular changes, particularly moving RBCs.

From the time courses, we observed the OMAG signal started to rise within 1 s, reached its peak in about 5 s once functional activation began and stayed at the top until activation ended. The IOSI reflectance signal started to grow gradually in about 1 s since the stimulation started and hit its maximum at ∼7th s. Such a difference in a time course might be explained by the fact that the IOSI signal was measured from the surface layer [Fig. 2(c)], while the OCT measurement was taken primarily from the deeper layer [Fig. 3(c)]. The spatial gradient of microvascular dilation delays was demonstrated by Tian et al.,^9^ where the vessel dilation was the fastest in deeper layers.

OMAG also revealed the laminar variation of hemodynamic response to neural activity over cortical depth. The results of our analysis showed that the largest response of OMAG signal to stimulation occurred in the layer IV of cortex. Our results agree with the previous studies on depth-dependent hemodynamic regulation where the most significant hemodynamic response was observed in the fourth cortical layer.^23^ The variability of hemodynamic response over cortical layers might be explained by the ability and necessity of brain to adapt the CBF locally to satisfy local metabolic demands,^7^ since every cortical layer has a different function (neural response, energy use, connectivity) and morphology.^24^

The OMAG signal is considered to be a product of moving particle velocity and its concentration. In other words, it is the total number of RBCs passing through the imaging voxel area per unit time, which is related to the concept of flux. Therefore, we hypothesize that the OMAG signal magnitude depends on blood flow velocity and hematocrit values. Choi et al.^25^ demonstrated that at 3.6 ms interval between adjacent B-scans, OMAG signal does not have strong relationship to RBC velocity, nor it is dependent on the size of vessels (vessel diameter). The reason is that for the time interval beyond 3 ms, the OMAG magnitudes tend to saturate for the flow velocities faster than 0.1  mm/s. However, it was found that increased particle concentration increases the OMAG signal intensity. Given that the time interval in our study was ∼3.3  ms and typical velocity for blood flow was faster than at least 0.3  mm/s, it is reasonable to assume that the velocity would saturate the OMAG signal, and the increase of OMAG signal from the mouse CBF during neural stimulation was attributable to the increase of RBCs, i.e., hematocrit. The *in vivo* study with laminar optical tomography^26^ suggests that a net increase in the linear density of RBCs in the capillaries accompanies the flow response to functional stimulus, which was explained by the increase of RBC stacking due to capillary dilation that occurs during stimulus response. The expansion of capillary diameter allows a higher plasma volume flow but is not sufficient to allow more than one RBC to pass at the same time, which results in an increased RBC stacking linear density. Thus, within a particular range of a time interval between consecutive B-scans, the OMAG signal has a potential to be effectively used as an indicator of blood flow flux change associated with the hemodynamic response.

A potential limitation of this study is that the measurements were performed in anesthetized animals. Different anesthetics have proven to disrupt the baseline (prestimulus resting) hemodynamics and functional response to stimuli. Ketamine was proven to induce the relaxation of cerebral blood vessels via their actions on the intracellular calcium dynamics of smooth muscle cells,^27^ which can potentially cause discrepancies in interpreting the experimental results of hemodynamic response. Nevertheless, all the compared measurements were performed under the same anesthesia conditions, which are expected to be subject to the same effect. We have developed procedures to evaluate the hemodynamics in a fully awake mouse,^28^ and the analysis of the results in awake animals is currently ongoing.

Overall, we believe that our findings provided an important opportunity to advance the understanding of hemodynamic events associated with the NVC. The OMAG technique we propose may prove to be a useful tool to investigate the depth-resolved variation of CBF in a fast way without any contrast agents required.

## 5 Conclusion

To conclude, the role of imaging techniques used to quantify the hemodynamics during functional activation is essential for studying the CBF adjustment at the NVC. In this paper, we demonstrated the development and application of a dual-modality imaging system composed of IOSI and OMAG. The results of our feasibility study showed that in addition to IOSI surface blood flow information, OMAG was able to provide complementary blood flow information at the depth of mouse somatosensory cortex. We believe that OMAG has a potential to enable the future investigation of depth-resolved CBF and to provide the insights of hemodynamic events associated with the NVC.

## Code, Data, and Materials Availability

All datasets generated and analyzed during the current study are available from the corresponding author on reasonable request.
